# Meeting individualized glycemic targets in primary care patients with type 2 diabetes in Spain

**DOI:** 10.1186/s12902-016-0090-1

**Published:** 2016-02-17

**Authors:** I. Miñambres, J. J. Mediavilla, J. Sarroca, A. Pérez

**Affiliations:** Department of Endocrinology and Nutrition Services, Hospital de la Santa Creu i Sant Pau, C/ Sant Quintí, 89, 08026 Barcelona, Spain; Research Institute, Hospital de la Santa Creu i Sant Pau, Barcelona, Spain; Centro de Salud Burgos Rural, Burgos, Spain; Almirall S.A., Barcelona, Spain; Department of Medicine, Universitat Autònoma de Barcelona (UAB), Barcelona, Spain; Diabetes and Metabolic Diseases CIBER (CIBERDEM), Barcelona, Spain

**Keywords:** Type 2 diabetes, Glycemic control, Glycemic targets, Individualization

## Abstract

**Background:**

Information about the achievement of glycemic targets in patients with type 2 diabetes according to different individualization strategies is scarce. Our aim was to analyze the allocation of type 2 diabetic patients into individualized glycemic targets according to different strategies of individualization and to assess the degree of achievement of adequate control.

**Methods:**

Cross-sectional analysis on 5382 type 2 diabetic patients in primary care setting in Spain between 2011 and 2012. Targets of HbA1c were assigned based on different strategies of individualization of glycemic targets: 1) the ADA/EASD consensus 2) The Spanish Diabetes Society (SED) consensus 3) a strategy that accounts for the risk of hypoglycemia (HYPO) considering the presence of a hypoglycemia during the last year and type of hypoglycemic treatment. Concordance between the different strategies was analyzed.

**Results:**

A total of 15.9, 17.1 and 67 % applied to ADA/EASD recommendation of HbA1c target of <6.5, < 7 and <8 % (48, 53 and 64 mmol/mol), and 31.9 and 67.4 % applied to the SED glycemic target of <6.5 and <7.5 % (<48 and 58 mmol/mol). Using the HYPO strategy, 53.5 % had a recommended HbA1c target <7 % (53 mmol/mol). There is a 94 % concordance between the ADA/EASD and SED strategies, and a concordance of 41–42 % between these strategies and HYPO strategy. Using the three different strategies, the overall proportion of patients achieving glycemic targets was 56–68 %.

**Conclusions:**

Individualization of glycemic targets increases the number of patients who are considered adequately controlled. The proposed HYPO strategy identifies a similar proportion of patients that achieve adequate glycemic control than ADA/EASD or SED strategies, but its concordance with these strategies in terms of patient classification is bad.

## Background

Recent clinical guidelines and expert committees on the management of type 2 diabetes have recommended individualization of glycemic targets based on patient characteristics, comorbid conditions, diabetes complications, duration of diabetes and risk of hypoglycemia [1–4]. The American Diabetes Association and European Association for the Study of Diabetes (ADA/EASD) [2] recommended a target HbA1c level <7 % (53 mmol/mol) for most patients with type 2 diabetes, however, a more relaxed target (HbA1c 7.5–8 % (58–64 mmol/mol)) should be aimed in patients with multiple comorbidities, reduced life expectancy, history of hypoglycemia, or advanced diabetes complications. On the other hand, a more stringent target such as HbA1c <6.5 % (48 mmol/mol) was considered beneficial in younger patients without comorbid conditions and with no adverse effects of antihyperglycemic treatment. Similarly, the national consensus from the Sociedad Española de Diabetes (SED – Spanish Diabetes Society) [3] recommended a stringent HbA1c target of <6.5 % (48 mmol/mol) in patients with newly diagnosed diabetes, age <70 years, and absence of diabetic complications, otherwise, a less stringent HbA1c goal of <7.5 % (58 mmol/mol) should be the target in the absence of these conditions. The American Association of Clinical Endocrinologists (AACE) also recommends the individualization of glycemic targets taking into account several factors that include concurrent illnesses and risk of hypoglycemia [5].

Despite the widespread acceptance of individualized glycemic control in patients with type 2 diabetes, information on the number of patients reaching these new recommended targets in different populations is scarce [6–8]. A recent analysis from the National Health and Nutrition Examination Survey (NHANES) revealed that about half of the US diabetic population would be considered inadequately controlled if a universal HbA1c target of <7 % (53 mmol/mol) was applied, compared with 30 % if using individualized ADA glycemic targets [6, 7]. In the analysis by Laiteerapong et al. [6], individualization of glycemic targets was performed taking into account the patient’s age, duration of diabetes, diabetes complications and significant comorbidities, but not the risk and the past history of hypoglycemia. Likewise, the study by Graciani et al. [8], conducted in 661 Spanish type 1 and type 2 diabetic patients, does not consider the risk of hypoglycemia, and information regarding diabetes complications was limited to cardiovascular disease and nephropathy. History of past hypoglycemia and insulin treatment are known and important predictors of a future hypoglycemic event [9–11], and, as stated in different recommendations, are important aspects to consider when assigning a patient to a certain HbA1c target.

In the Spanish type 2 diabetic population included in the Diabcontrol Study [12], we analyzed the distribution of patients within the individualized glycemic targets recommended by the ADA/EASD and the SED consensus and according to an original strategy that considered risk of hypoglycemia. Furthermore, we compare the different strategies of individualization of glycemic targets and their concordance and provide information concerning the degree of achievement of an adequate glycemic control.

## Methods

### Study design

Patients included in this analysis participated in the Diabcontrol Study previously described [12]. Briefly, this was an epidemiological, cross-sectional study conducted in primary care centers throughout Spain between 2011 and 2012, which included 5382 patients with type 2 diabetes receiving antidiabetic treatment. All patients completed a single clinical visit in which HbA1c was measured in capillary blood (A1CNow+) [13].

We identified clinical variables used in the ADA/EASD [2] and SED consensus [3]. Young age was considered ≤70 and ≤75 years for the SED and ADA/EASD strategies, respectively, whereas long duration of diabetes was defined as > 10 years for both strategies. The presence of micro- and macro-vascular disease was extracted from the clinical database and included the presence of macroalbuminuria (urine albumin-creatinine ratio of >300 mg/g), glomerular filtration rate < 60 ml/min/1.73 m^2^, diabetic foot, diabetic retinopathy, polyneuropathy, peripheral vascular disease, cerebrovascular disease and coronary vascular disease. We also reviewed information on antihyperglycemic treatment and history of a hypoglycemia that required medical assistance during the 12 months prior to the study visit. Patients with missing data concerning any variable necessary for patient classification were not included in the analysis.

Table 1 shows the strategies for assigning patients with type 2 diabetes into different categories of HbA1c: 1) ADA/EASD strategy considered patient’s age, duration of diabetes and presence of advanced micro- and macro-vascular complications; 2) SED strategy considered patient’s age, duration of diabetes and the presence of advanced micro- and macro-vascular complications; and 3) The HYPO strategy considered the type of hypoglycemic treatment and the need for medical assistance due to an hypoglycemic event during the 12 months prior to the study visit. In order to determine the concordance between the three strategies, each one was simplified into two categories named low and high risk. In addition, we evaluated the impact of taking into account hypoglycemia risk in the ADA/EASD and SED strategies. Patients with a previous hypoglycemic episode or treated with two or more insulin doses were directly classified into highest risk categories from the ADA/EASD and SED consensus.

**Table 1:** Strategies for individualization of glycemic targets

Category name	HbA1c target	Low/high risk	Patient characteristics				
			Treatment	Previous hypoglycemia	Age	Duration of diabetes	Diabetes complications^a^
ADA/EASD- 1	<6.5	Low	Any	Any	Any	<5	No
ADA/EASD-2	<7	Low	Any	Any	≤75	5-9	No
ADA/EASD-3	<8	High	Any	Any	>75	Any	Any
					Any	>10	Any
					Any	Any	Yes
SED-1	≤6.5	Low	Any	Any	≤70	<10	No
SED-2	≤7.5	High	Any	Any	>70	Any	Any
					Any	>10	Any
					Any	Any	Yes
HYPO-1	<7	Low	Not insulin, not SU, not glinides	No	Any	Any	Any
HYPO-2	<7.5	Low	Basal insulin, SU, or glinides	No	Any	Any	Any
HYPO-3	<8	High	Insulin (≥2 doses)	Any	Any	Any	Any
			Any	Yes	Any	Any	Any

^a^Diabetes complications for ADA/EASD and SED categories were considered to be macroalbuminuria (urine albumin-creatinine ratio of >300 mg/g), chronic kidney disease (CKD), diabetic foot, diabetic retinopathy, polyneuropathy, peripheral vascular disease, cerebrovascular disease or coronary vascular disease

SU: sulphonylureas

The study was approved by the Unitat d’Avaluació, Suport i Prevenció (UASP) of Hospital Clínic in Barcelona and was conducted according to the principles of the Declaration of Helsinki and Good Clinical Practice standards. We obtained written informed consent from all patients before their inclusion in the study.

### Statistical analysis

The results were expressed as frequencies and percentages for qualitative variables and as mean and standard deviation for quantitative variables. Missing data can be inferred by the total number of patients included in each analysis. Cohen’s kappa coefficient was used to evaluate the concordance between the different strategies of patient classification. All statistical tests were considered significant at *p* < 0.05. Statistical analysis was performed with the SAS statistical package (version 9.3).

## Results

### Patient characteristics

A total of 5382 individuals with type 2 diabetes were included in the study. Patient characteristics and hypoglycemic treatment are shown in Table 2. The mean age was 66.7 years, and 53 % were men. Diabetes complications were present in 43.6 % of the population. Average HbA1c was 7.3 ± 1.2 % (56 ± 9.2 mmol/mol) and 48.6 % of the patients had an HbA1c of < 7 % (53 mmol/mol).

**Table 2:** Patient characteristics and hypoglycemic treatment

Sex (% men)		53
Age (years)		66.7 ± 10.8
BMI (kg/m^2^)		29.9 ± 5
Duration of diabetes (years)		8.8 ± 6.3
HbA1c (%/mmol/mol)		7.3 ± 1.2/56 ± 9.2
Diabetes complications (%)		43.6
Macrovascular		23
Microvascular		23.5
Hypoglycemic treatment (%)	Oral agents (OA) only	77.7
	•Sulphonylureas or glinides	26.7
	Insulin with/without OA	22.3
	•≥2 doses	9.3
Previous hypoglycemia that required medical assistance during the last year (%)		6.8

Data expressed as Mean ± SD/% of total

BMI: body mass index

### Individualization of glycemic targets based on ADA/EASD consensus (*n* = 5267)

Using the ADA/EASD consensus and including information regarding patients’ age, duration of diabetes and the presence of diabetic complications, a target HbA1c of < 6.5 % (48 mmol/mol) applied to 15.9 % of the study population, while the conventional target of < 7 % (53 mmol/mol) applied to 17.1 %. On the other hand, a less stringent target (HbA1c < 8 % (64 mmol/mol)) applied to 67 % of our type 2 diabetic patients. According to these individualized glycemic targets, 67.4 % of our population was considered adequately controlled.

When antihyperglycemic treatment and the presence of a hypoglycemia during the last year were included in the ADA/EASD strategy for individualization of HbA1c targets, the percentage of patients corresponding to a more stringent, conventional and less stringent target were 14.9, 15.5 and 69.6 %, respectively. According to these criteria, 68.5 % of the patients were considered adequately controlled. The level of concordance between the two risk stratification strategies is 97.5 % (kappa coefficient 0.9413; data not shown).

### Individualization of glycemic targets according to the SED guidelines (*n* = 5267)

Using the SED consensus, an HbA1c target of < 6.5 % (48 mmol/mol) applied to 31.9 % of the study population, whereas a less stringent target of < 7.5 % (58 mmol/mol) applied to 68.1 %. If these individualized glycemic targets were considered, 55.2 % of our patients would be adequately controlled. If patients with a previous history of hypoglycemia that required medical assistance during the last year and/or patients taking ≥ 2 insulin injections were directly considered within the SED-2 category, the percentages of patients corresponding to the more stringent and the less stringent target would be 29.4 and 70.6 %, respectively. If these targets were used, 56 % of our patients would be adequately controlled. The level of concordance between the two risk stratification strategies is 97.5 % (kappa coefficient 0.9403; data not shown).

### Individualization of glycemic targets based only on antihyperglycemic treatment and the presence of hypoglycemia during the last year (*n* = 5304)

When we stratified patients according to antihyperglycemic treatment and the presence of hypoglycemia during the last year, as shown in Table 1, 53.5 % were considered to have a low risk of hypoglycemia, and therefore corresponded to a target of HbA1c < 7 % (53 mmol/mol). A target of HbA1c < 7.5 % (58 mmol/mol) applied to 32.6 % of the population and a less stringent target of HbA1c < 8 % (64 mmol/mol) applied to 13.9 %. When using this simple strategy of individualizing glycemic targets, 60.7 % of the population was considered to have an adequate glycemic control according to the capillary HbA1c (A1CNow+) measured at the single study visit.

### Concordance between the different strategies of individualizing glycemic targets

The level of concordance between the ADA/EASD strategy and the SED consensus is very good, whether or not they include information regarding past history of hypoglycemia and diabetes treatment, with a concordance of 94 and 94.3 %, respectively (Table 3). However, the level of concordance between the strategy based only on antihyperglycemic treatment and history of hypoglycemia (HYPO) and the ADA/EASD and SED strategies was 42 and 41 %, respectively (Table 4).

**Table 3:** Concordance between the ADA/EASD and SED strategies in terms of patient classification

	SED			
ADA/EASD	Low Risk (n(%))	High Risk (n(%))	Total (n(%))	P^a,b^
Low Risk	1517 (30.3)	91 (1.8)	1608 (32.1)	<0.0001
High Risk	211 (4.2)	3195 (63.7)	3406 (67.9)	
Total	1728 (34.5)	3286 (65.5)	5014 (100)	
	SED + HYPO			
ADA/EASD + HYPO	Low Risk (n(%))	High Risk (n(%))	Total (n(%))	P^a,c^
Low Risk	1395 (27.8)	85 (1.7)	1480 (29.5)	<0.0001
High Risk	200 (4)	3334 (66.5)	3534 (70.5)	
Total	1595 (31.8)	3419 (68.2)	5014 (100)	

^a^Chi Square test

^b^Kappa Coefficient 0.8644

^c^Kappa Coefficient 0.8664

**Table 4:** Concordance between the risk of hypoglycemia strategy (HYPO) and the ADA/EASD and SED strategies in terms of patient classification

	HYPO			
ADA/EASD	Low Risk (n(%))	High Risk (n(%))	Total (n(%))	P^a,b^
Low Risk	1583 (30.5)	134 (2.5)	1717 (33)	<0.0001
High Risk	2282 (55.5)	595 (11.5)	2877 (67)	
Total	3865 (86)	729 (14)	4595 (100)	
	HYPO			
SED	Low Risk (n(%))	High Risk (n(%))	Total (n(%))	P^a,c^
Low Risk	1463 (29.5)	128 (2.5)	1591 (32)	<0.0001
High Risk	2804 (56.5)	572 (11.5)	3376 (68)	
Total	4267 (86)	700 (14)	4967 (100)	

^a^Chi Square test

^ b^Kappa Coefficient 0.0662

^c^Kappa Coefficient 0.0616

## Discussion

The individualization of glycemic targets using different recommended strategies, such as the ADA/EASD strategy used by Laiteerapong et al. [2, 6] or the SED consensus strategies [3], reveals that 56–68 % of Spanish diabetic patients receiving pharmacological treatment are under adequate metabolic control. A similar proportion of patients with adequate glycemic control was obtained when individualization of targets were based only on the risk of suffering a hypoglycemic episode. We found a close agreement on the concordance of ADA/EASD and SED strategies with regards of patient classification; however, the concordance between these strategies and the HYPO strategy that is based on the risk of hypoglycemia was low. Of interest, the inclusion of a history of hypoglycemia and type of hypoglycemic treatment into the ADA/EASD and SED strategies does not modify patient classification into different glycemic targets.

Using the conventional HbA1c target level of < 7 % (53 mmol/mol), 48.6 % of the Spanish type 2 patients under pharmacological treatment at primary care centers are adequately controlled. Previous studies conducted in Spanish patients reported a mean HbA1c between 7 and 7.5 % (53 and 58 mmol/mol) with 40–56 % of patients achieving HbA1c < 7 % (53 mmol/mol) [14–19]. Differences between our findings and previous studies can be explained in part by the fact that in this study all patients were receiving pharmacological treatment, while in some previous studies 23 % of patients were treated with diet only and 77 % of patients were treated with antidiabetic agents [16–18]. Furthermore, in the absence of an integrated care system for diabetes management, variations in diabetes management among the different primary care settings may have contributed [20].

Recent studies have questioned whether the conventional target of HbA1c 7 % (53 mmol/mol) for defining adequate glycemic control is suitable for all type 2 diabetic patients. The relationship between glucose control and the development of micro- and macro-vascular complications of diabetes is well established [21]. Results from the UKPDS revealed that glycemic control prevented microvascular complications; however, intensive glucose control had minor effect in the development of macrovascular complications [22]. Several trials in patients with type 2 diabetes have investigated the effects of intensive glucose lowering on cardiovascular events. The ACCORD (Action to Control Cardiovascular Risk in Diabetes) trial had to be stopped because of an increase in all-cause and cardiovascular mortality in the intensive glycemic control group [23]. Two other trials, ADVANCE (Action in Diabetes and Vascular Disease) [24] and VADT (Veterans Affairs Diabetes Trial) [25], reported weight gain and an increase in the rate of hypoglycemia with no benefit in terms of preventing macrovascular complications in the intensive glucose management arms. These findings have led to the development of several consensus documents on the individualization of glycemic targets according to patient’s characteristics [1–3]. As the individualization of glycemic targets is a new concept in diabetes care, information regarding the degree of glycemic control according to these new targets is very limited. Laiteerapong et al. have recently published data about the degree of glycemic control in type 2 adult diabetic patients in the US and find that 70 and 71 % of the population would be adequately controlled according to the ADA guidelines and the Ismail-Beigi strategy, respectively. In order to make results comparable, we have stratified our population of Spanish type 2 diabetic patients according to the similar criteria of the ADA/EASD consensus used by Laiteerapong et al. and have found that 67.4 % of our patients can be considered adequately controlled, a very similar proportion to the US population, especially if we consider the greater difficulty of achieving targets in patients with pharmacological treatment [17, 26].

The presence of a previous episode of hypoglycemia as well as the choice of antidiabetic therapy, in particular in patients receiving complex insulin regimens, has consistently been reported as a strong predictor of hypoglycemia [9–11]. Actually, some of the strategies for individualization of glycemic targets including the ADA/EASD consensus [2] and the Ismail-Beigi strategy [1] take this information into account when assigning patients to a certain HbA1c target. In our analysis, the inclusion of information on hypoglycemia and treatment with ≥ 2 insulin injections/day as risk markers of hypoglycemia risk in the ADA/EASD strategy results in similar percentages of patients corresponding to each HbA1c target. Although the SED strategy does not take into account the patient’s risk of hypoglycemia, when we included the same markers of hypoglycemia risk into the highest risk category, the concordance with the classical SED classification is also very good. This led us to believe that taking into account information regarding patients’ risk of hypoglycemia may represent a simple and feasible way of assigning patients to different HbA1c targets. However, our results show a low degree of concordance with previously described strategies in terms of assigning patients to pre-specified glycemic targets (41–42 % of concordance with kappa coefficients < 0.1). As shown in Table 4, 55.5 and 56.5 % of patients were classified into highest risk categories according to the ADA/EASD and SED consensus but into low risk category according to the HYPO strategy; this explains the low degree of concordance between the different strategies.

Several limitations of this study should be noted. We only considered two recommended strategies for individualization of glycemic targets among the amount of them published and there is a lack of information concerning different psychosocial and economic contexts, factors included in the ADA/EASD strategy [2]. It should also be noticed that, HbA1c was measured by a capillary method (A1CNow+), which although not being standardized, it has a good correlation with NGSP standardized techniques [13].

Strengths of this study include the fact that clinical information is collected from a primary care clinical database, excluding possible bias from self-reporting. Furthermore, we add information regarding past history of hypoglycemia and severe retinopathy or diabetic foot, which could not be included in the study by Laiteerapong et al. [6] and we use the same method of HbA1c determination in every patient. Finally, it should be noted that our study sample was recruited between 2011 and 2012, while the population sample included in the US analysis was recruited between 2007 and 2008. So, although the adoption of guideline recommendations for individualization is slow and ongoing, our analysis may reflect not only the degree of control of our population, but also how professionals are applying the new recommendations.

## Conclusion

In conclusion, our results indicate that the new recommendations for implementation of individualized glycemic targets lead to a higher number of patients that can be considered to have adequate glycemic control. The ADA/EASD and SED strategies of individualizing glycemic targets are in high degree of concordance, with 67–68 % of patients that correspond to a less stringent target than the conventional HbA1c < 7 % (53 mmol/mol). However, this should not lead to complacency, since 30–45 % of patients still fail to meet the required targets. Long-term studies are needed to determine if the new strategy recommendations will lead to a reduction of diabetic complications and hypoglycemia in patients with diabetes. Waiting for these results, all current strategies are important but incomplete and need to be complemented by a clinical judgment to determine effective and safe glycemic targets.
